# Use of O-arm to place an intrathecal catheter through a bony fusion mass: case report

**DOI:** 10.3389/fresc.2025.1530801

**Published:** 2025-05-22

**Authors:** Francesco Sammartino, Sheital Bavishi, Brian Dalm

**Affiliations:** ^1^ Department of Physical Medicine and Rehabilitation, The Ohio State University, Columbus, OH, United States; ^2^ Department of Neurological Surgery, The Ohio State University, Columbus, OH, United States

**Keywords:** spasticity, spinal cord injury, O-arm, navigation, intrathecal baclofen

## Abstract

**Background:**

Intrathecal baclofen (ITB) delivery is an FDA-approved indication for patients with intractable spasticity. Often, implantation in these patients can be considerably challenging, especially if previous surgical fusion involves the procedure access location.

**Case report:**

We present the case of a 27-year-old female with T2 American Spinal Injury Association (ASIA) A spinal cord injury (SCI) and chronic spastic dystonia. She was maximized on oral medications without satisfactory control of her painful muscle spasms and was a candidate for ITB trial, which ultimately failed due to the difficulty of accessing the spinal canal due to extensive pseudoarthrosis secondary to thoracic to lumbar fusion. A decision was made to directly implant the pump in the operative room using O-arm–aided neuronavigation to guide catheter access at L5–S1. Currently, at 22 months of follow-up post-pump implant, ITB delivery has led to persistent improvements in her spastic dystonia and many aspects of quality of life.

**Discussion:**

The current case indicates that a multidisciplinary approach when considering surgical treatments for medication-refractory spasticity may help expand the indications to large numbers of patients with postsurgical spine abnormalities.

## Introduction

Baclofen, a GABA agonist and the most widely used antispasmodic agent, reduces spasticity through a dual mechanism in the spinal cord. Presynaptically, it inhibits calcium uptake, thereby reducing the release of excitatory neurotransmitters. Postsynaptically, at higher cerebrospinal fluid (CSF) concentrations, it can also antagonize the effects of these same excitatory neurotransmitters (1). The limited lipid solubility of baclofen restricts its passage across the blood–brain barrier. As a result, oral administration, even at high dosages, yields relatively low cerebrospinal fluid concentrations, leading to suboptimal inhibition of spasticity (2). The management of spasticity in patients with spinal cord injury (SCI) that is not responsive to the combination of oral medications and targeted toxin injections poses an immense therapeutic challenge. Often, those patients are considered for advanced interventional therapies such as intrathecal drug delivery systems (IDDS). Intrathecal pumps were originally approved for patients with metastatic diseases and those with intractable noncancer medical conditions that cause severe chronic pain. Currently, intrathecal baclofen (ITB) is used to treat severe spasticity that doesn't respond to oral medications or causes unacceptable side effects. Careful evaluation is needed for patients with low muscle tone, hydrocephalus, bulbar dysfunction, inadequate body mass, or cognitive deficits (3). The most common disease indications include cerebral palsy and spinal cord and brain injury (4). More recently, the improvement of hardware and surgical techniques has allowed the expansion of intrathecal baclofen use in patients with dystonia, amyotrophic lateral sclerosis, Friedreich's ataxia, stiff-man syndrome (5), and central pain syndromes associated with spasticity (6). Although there remains some debate on the long-term safety concerning the use of IDDS in patients with treatment-resistant spasticity (7), these devices are beneficial, as demonstrated in numerous reports. However, implantation can be challenging especially if a pre-existent surgical fusion involves the procedure access location.

## Case presentation

A 27-year-old woman presented to the clinic with chronic spastic paraparesis, following a motor vehicle accident she had at the age of 7. At that time, she received acute care in a local hospital followed by inpatient rehabilitation. Her initial injury was classified as “T2—complete.”

**Figure 1:**
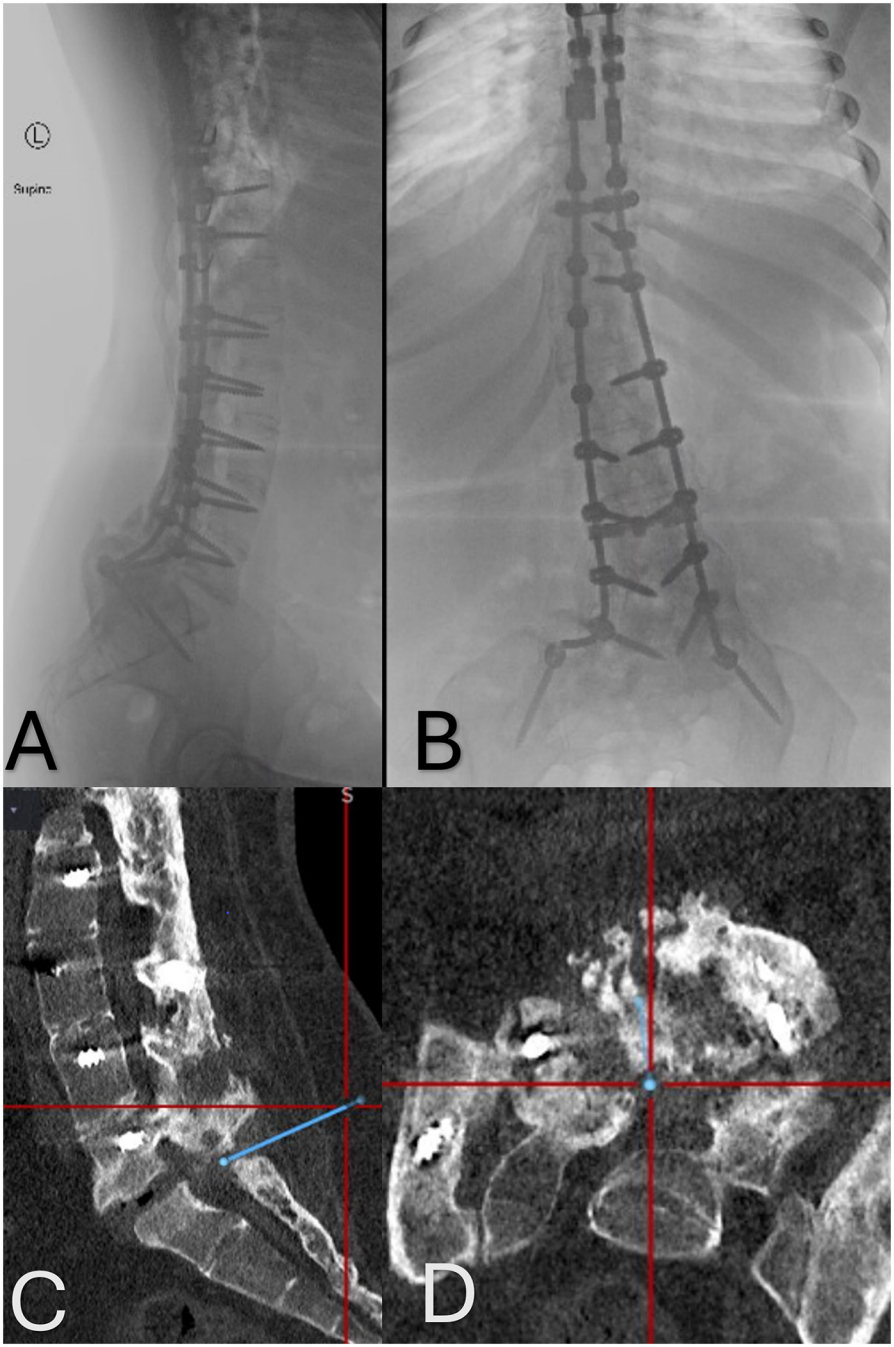
**(A,B)** Anteroposterior and lateral x-rays of the thoracolumbar spine demonstrating the extensive instrumentation utilized to stabilize the patient's spine. **(C,D)** Sagittal and axial CT images showing the significant bony fusion mass and the small area of pseudoarthrosis at the L5–S1 level. The blue line indicated the planned trajectory used for the placement of the Touhy needle into the intrathecal space.

After undergoing emergent T1–T6 stabilization surgery, she developed scoliosis, which was followed by a revision surgery 5 years later with extension of the fusion to the pelvis (Figures 1A,B).

More recently, she started having worsened spasms in the last year, concomitant with a change in bowel function. She also complained of neck and right shoulder pain. She had significant clonus, despite 80 mg of oral baclofen daily and scheduled 500 mg methocarbamol TID without resolution. She also had bladder [genitourinary (GU)] spasms and received botulinum toxin injections into her bladder from urology.

On exam, she presented intact strength and sensation to bilateral upper limbs, a sensory level at T2 with no movements below the level of injury. She had bilateral hip abductor tone Ashworth 1/4, left knee flexor 1/4, right knee flexor 1/4, left knee extensor 1/4, right knee extensor 1/4, left plantar flexor ¼, and right plantar flexor 1/4 with immediate bilateral lower lim (BLL) spasms with any range of motion (ROM) that involved her back muscles. She was not able to tolerate further increase in her oral baclofen dosing, and methocarbamol dosing was not increased as it was ineffective.

After having a discussion about intrathecal baclofen pump placement and its potential effects on her dystonia, she was referred to the interventional pain team for an intrathecal baclofen trial. Review of thoracic and lumbar magnetic resonance imaging revealed no significant stenosis that would preclude the advancement of an intrathecal catheter; however, the lumbar magnetic resonance images were largely noninterpretable because of the artifacts from instrumentation at the L1–L4 region. Spine CT demonstrated the presence of a large fusion mass going all the way down to L5. There was a pseudoarthrosis at L5–S1 with a small gap in the posterior fusion mass thought to be used as a potential access to the intrathecal space at L5–S1. However, the intrathecal space could not able to be accessed under fluoroscopy guidance to complete the trial.

Although the trial was not successful, a decision was made to proceed with the pump and catheter implantation by using a three-dimensional (3D) image guidance system, the Stealth Image Guidance System (Medtronic Inc., Minneapolis, MN, USA), in combination with the O-arm (Medtronic). The O-arm is a cone beam computed tomography (cbCT) device that allows for the acquisition of an intraoperative cbCT scan, which automatically registers to the image-guided system. Using infrared technology, the image-guided system can track instruments to navigate the spine from the intraoperative cbCT scan.

With an image-guided probe placed on the skin, the bony fusion mass at the L5–S1 level was visualized. This region was optimal for access because of a gap in the fusion mass identified on the prior CT (Figures 1C,D). After performing a small linear skin incision, the intraoperative navigation system was used to guide a 15 G Touhy needle into the fusion gap and into the intrathecal space at L5–S1. Cerebrospinal fluid was obtained on the first pass. Next, a 2.2-mm-diameter intrathecal catheter was advanced cephalad in the intrathecal space through the Tuohy needle.

Fluoroscopy confirmed the position of the catheter at T9–T10 interspace. The catheter was secured with an anchor and silk sutures at the fascial level. The rest of the procedure for tunneling the catheter and placement of the pump in the abdominal pocket proceeded in a standard manner. The pump reservoir had been filled with 20 ml of baclofen 500 mcg per ml and was programmed to deliver 25 mcg over 24 h.

The patient was discharged from the hospital the following day and was followed up in the clinic 10 days later. At the first visit, a decision was made to start decreasing the oral baclofen to 20 mg TID while increasing the daily dose of intrathecal baclofen to 50.04 mcg/24 h. In the follow-up appointment 1 week later, her oral baclofen was further decreased to 20 mg BID, and the intrathecal baclofen increased to 74.98 mcg/day. In the following week, she was weaned completely off the oral baclofen, and by 2 months later, the baclofen concentration was changed to 2,000 mcg/ml, and the pump had been reprogrammed to deliver 125.32 mcg/day by that time.

At her last follow-up 22 months after the pump placement, she continues to benefit from the intrathecal baclofen and remains off the oral baclofen and the methocarbamol. Her current daily dose of baclofen remains stable at 125.32 mcg/day, with Ashworth 0 scores in the lower limbs. She is able to work sitting in her chair for up to 12 h per day without spasms affecting her working ability.

## Discussion

The authors present a novel method of intrathecal catheter placement through a solid bony fusion mass utilizing intraoperative real-time 3D image guidance. The relevance of this case lies in showing how image guidance can enable difficult spinal canal access while minimizing patient discomfort and facilitating care.

This patient likely presented with spastic dystonia, a condition that is a much less well understood entity, which is characterized by the presence of a tonic muscle contraction in the absence of voluntary movement or spinal reflex activation which is thought to derive from an increased baseline excitability of the alpha-motoneurons.

Spastic dystonia is seen most frequently in the upper limb, but it can also be seen during standing and gait where the subject may adopt a posture with plantarflexion and/or inversion at the ankle, toe flexion, pronounced extension at the knee which limits functional mobility. In a recent study (8), the authors found that approximately 27% of MS patients in their cohort had spastic dystonia instead of spasticity, thus suggesting that this condition is likely underdiagnosed. Currently, the diagnosis of spasticity relies heavily on the subjective assessment of velocity-dependent hypertonia, a measure that lacks specificity as it doesn't distinguish between neural and non-neural contributions to increased muscle tone. This distinction is particularly relevant because if velocity-dependent tone is mainly due to intrinsic hypertonia, pharmacological and neurosurgical interventions, such as baclofen or selective dorsal rhizotomy, are usually more effective in addressing spasticity. Unfortunately, the only true method of quantifying the spasticity component is to measure tonic stretch reflexes using EMG.

In this report, the patient's pattern of spasticity was mainly characterized by a dynamic increase in tone, which benefited greatly from intrathecal baclofen delivery followed by fine-tuned programming. In this case, the intrathecal baclofen concentration was 500 mcg/ml, as recommended by the ITB Therapy Best Practices Expert Consensus Panel (9), and the pump was programmed to deliver 25 mcg of baclofen over 24 h, which is lower than a typical adult screening dose, given that the patient was not able to have a preoperative baclofen trial and her pattern of spasticity was not typical.

The decision to bypass the trial phase is controversial. A recent survey (10) demonstrated that approximately 70% of providers almost always perform a screening test with intrathecal baclofen before proceeding to implantation. Interestingly, the choice of not performing the trial seemed to be linked to the specialty. In this study, the failure to access the lumbar intrathecal space under fluoroscopy and the presence of ongoing lower limbs disabling “dynamic” spasticity, which was not effectively controlled by oral baclofen, were the main reasons to skip the screening test. Other reasons from the literature include some physicians believing that trialing does not provide a reliable indication of long-term therapeutic outcome and that trialing increases the risks of infection (9).

The treatment of generalized dystonia is often difficult and frustrating for both patient and physician. Oral medications used to treat generalized dystonia include baclofen, clonazepam, trihexyphenidyl, and levodopa. In general, oral medications alone are only effective on dystonia in a minority of patients and cause dramatic improvement in even fewer. From this evidence, ITB appears to be the treatment of choice for severe generalized dystonia, particularly secondary dystonia, which is inadequately treated by oral medications. While alternative methods for CNS baclofen delivery, such as intraventricular (11) or prepontine cistern (12) catheter placement, have been described and could benefit patients with challenging anatomy, a more conventional lumbar spine access was preferred in this particular case.

During the presurgical case evaluation, two options were discussed: one was to directly drill through the fusion mass to place the catheter, and the second was to attempt to place the catheter between the small opening at L5–S1. Drilling the mass was excluded because it could have led to a higher chance of pseudomeningocele development. The decision was made to proceed with the second option and attempt to place the needle with intraoperative navigation of the needle to maximize the chance of accessing the correct space.

A number of different approaches have been described to safely access the lumbar intrathecal space, as often patients with severe spasticity have comorbid neuromuscular scoliosis (13), and both spinal torsion and instrumented spinal fusion constructs complicate catheter insertion at this site. While techniques such as drilling through the fusion mass, K-wire navigation, and cervical or foramen magnum catheter insertion have been described, they are limited by the potential for CSF leakage, catheter dislodgement, and adjacent neural injury (14). To note, the risk of pseudomeningocele has been reported in the literature as high as 25% in a large retrospective series (15), and dystonia in particular could make sealing of the dural puncture at the catheter entry site slower or less stable. More recently, the advent of intraoperative 3D imaging has increased the accuracy of pedicle screws placement in deformed spines (16) with less radiation dose delivered to the surgeon compared with conventional fluoroscopy.

Intraoperative image guidance is an effective tool for the placement of spinal instrumentation and is commonly used in Neurosurgery. In this case, it was particularly advantageous, allowing for accurate navigation of the narrow target used to place the intrathecal catheter with limited surgical morbidity. Without the Stealth image guidance system, a larger open dissection and exploration of the fusion mass would have been required, potentially increasing the surgical risk and patient morbidity. Another advantage of using 3D image guidance is that radiation exposure to the patient is lower than that of standard C-arm fluoroscopy. Radiation exposure to the surgeon and surgical staff has been reported at or near zero when using this technology for spinal fusion procedures.

Limitations to the study are mainly related to the lack of extensive long-term follow-up and the limited generalizability of a single case report. In the literature, the complication rate following baclofen intrathecal pump surgery is difficult to establish, as there are several differences between methods of implantation across centers. In a recent single-center review on over 400 patients (14), the authors reported that 9.3% experienced an infection, 4.9% a CSF leak, 15.1% a problem with the catheter, and 1% a problem related to the pump. In their experience, catheter problems were the most common complication and occurred more frequently during the first year after the implant. In a similar fashion, the rate of long-term (over 10 years from the implantation surgery) complications is highly variable between centers. In a recent review (7), the authors reported a range of complications requiring surgical measures with an annual frequency ranging from 10.9% to 49.4%. The infection rate per year was between 0.7% and 5.2%, which is overall considered reasonable compared with other neurosurgical procedures.

In summary, this case demonstrates the possibility of placement of intrathecal baclofen pump and catheter in challenging conditions characterized by abnormal anatomy such as large fusion masses and pseudoarthrosis, by using intraoperative real-time 3D imaging. In addition, this case highlights the potential contribution of ITB therapy to improve the quality of life of patients with spastic dystonia following SCI.

## Data Availability

The raw data supporting the conclusions of this article will be made available by the authors, without undue reservation.
